# Methods of behavioral testing in dogs: a scoping review and analysis of test stimuli

**DOI:** 10.3389/fvets.2024.1455574

**Published:** 2024-10-16

**Authors:** Ariella Y. Moser, Mitchell Welch, Wendy Y. Brown, Paul McGreevy, Pauleen C. Bennett

**Affiliations:** ^1^School of Environmental and Rural Science, University of New England, Armidale, NSW, Australia; ^2^Anthrozoology Research Group, School of Psychology and Public Health, La Trobe University, Bendigo, VIC, Australia; ^3^School of Science and Technology, University of New England, Armidale, NSW, Australia; ^4^Sydney School of Veterinary Science, University of Sydney, Sydney, NSW, Australia

**Keywords:** behavioral assessment, behavioral testing, canine, dog cognition, dog personality, qualitative analysis, scoping review, temperament testing

## Abstract

**Background:**

Behavioral testing is widely used to measure individual differences in behavior and cognition among dogs and predict underlying psychological traits. However, the diverse applications, methodological variability, and lack of standardization in canine behavioral testing has posed challenges for researchers and practitioners seeking to use these tests. To address these complexities, this review sought to synthesize and describe behavioral testing methods by creating a framework that uses a “dog-centric” perspective to categorize the test stimuli used to elicit responses from dogs.

**Methods:**

A scoping review was conducted to identify scientific literature that has reported behavioral testing to assess psychological traits in dogs. Five online databases were systematically searched. Following this, an inductive content analysis was conducted to evaluate and summarize the behavioral testing methods in the literature.

**Results:**

A total of 392 publications met the selection criteria and were included in the analysis, collectively reporting 2,362 behavioral tests. These tests were individually evaluated and categorized. Our content analysis distinguished 29 subcategories of behavioral testing stimuli that have been used, grouped into three major categories: human-oriented stimuli; environmental stimuli; and motivator-oriented stimuli.

**Conclusion:**

Despite the methodological heterogeneity observed across behavioral testing methods, our study identified commonalities in many of the stimuli used in test protocols. The resulting framework provides a practical overview of published behavioral tests and their applications, which may assist researchers in selecting and designing appropriate tests for their purposes.

## 1 Introduction

Behavioral testing offers an empirical lens to reveal individual differences in the behavior and cognition of animals (1–3), including dogs (4, 5). Domestic dogs exhibit substantial variation in their behavioral tendencies and cognitive abilities. This can be seen, for example, in the diversity of behavioral phenotypes that characterize dog breeds (6–9). Even within a given breed, individual dogs exhibit extensive variation in their behavior and cognition (9–11). Accordingly, the attributes of individual dogs need to be considered and assessed to predict their behavior as companions and co-workers.

A behavioral test is a standardized protocol that presents a stimulus designed to elicit a measurable response in a subject. In many cases, a battery of behavioral tests, employing a series of stimuli, is used [e.g., (12, 13)]. The responses of different dogs undergoing the same protocol can then be compared to reveal individual differences. The premise of behavioral testing is that these responses reflect underlying traits and, in doing so, predict behavior beyond the testing context (5). In animal behavior research, “traits” are the inter-individual differences in behavior that are relatively stable across time and contexts (14). The term is most often used in relation to personality [e.g., (15)], but has also been used to describe perceived cognitive abilities or tendencies [e.g., (16)]. As these are types of psychological difference that we attempt to infer from dogs’ behavior, here we will refer to these as “psychological traits”.

Historically, canine behavioral tests have been used as a research tool in fields that include psychology [e.g., (17, 18)], neurophysiology [e.g., (19, 20)], animal science [e.g., (21, 22)] and ethology [e.g., (23, 24)], to answer questions about the biological and environmental bases of individual differences in the behavior of humans and animals. Beyond the scientific research context, canine behavioral tests have had many practical applications. For example, they are used to assess working dog candidates for various roles [for review, see (25, 26)], to determine dogs’ suitability for adoption from shelters [for review, see (27–29)], and to determine dogs’ breeding suitability according to breed club standards [e.g., (30, 31)]. Unsurprisingly, given this diverse range of applications, there is a great deal of heterogeneity in the behavioral testing literature concerning the studied populations, characteristics, interpretations, and methodologies that have been used.

Behavioral tests are often designed to reveal a certain trait (e.g., laterality, sociability), or super-trait (e.g., boldness) but may do so only imperfectly. Despite efforts to devise frameworks that define and categorize canine traits (25, 32–34), canine science has so far resisted the uptake of standard terminology or shared definitions. This may, in part, reflect the disparity of reasons for undertaking canine behavioral research (35). An additional problem is that we cannot measure psychological traits directly. Instead, we can measure only behavioral and physiological responses and then infer their meaning. Such inferences are primarily made in two ways: (1) *a priori* expectations that a test is designed to measure a certain trait (e.g., use of a loud sound intending to reveal ‘noise sensitivity’); and (2) *post hoc* interpretations, often from grouping correlated variables with factor analysis and using a descriptive label for each factor (e.g., cowering, vocalization, and distance from and latency to approach an unfamiliar human might be labelled ‘fearfulness’). Unfortunately, any attempt to interpret behavior is vulnerable to subjectivity and there is no consensus on the emergent terminology nor on the number of psychological dimensions that exist in dogs (4, 5, 36).

There is also currently no established methodological standard to adhere to when conducting behavioral tests. Protocols for tests and test batteries may be entirely or partly original or they may be adapted, rearranged, or replicated from previously established tests. In addition, data collection methods differ considerably and can range from subjective ratings of behavior, ratings of behavior on a scale, and behavior coding, to physiological measures or data from sensors (e.g., accelerometers, infrared beam breakers). This diversity in methodology gives rise to inconsistency among the tests’ degrees of standardization, reliability, and validity [for detailed discussions on assessing these qualities, see (1, 4, 5, 29, 36)]. Given the many diverse aims of behavioral tests and continuously evolving methods arising from nascent ideas and emergent technologies, much of this inconsistency has been unavoidable.

The current state of flux concerning terminology and methodology in canine behavioral testing has frustrated attempts to unify or compare findings (4, 5, 36, 37). That said, there are similarities among many behavioral testing protocols when these are considered from the perspective of the dog being tested. From this “dog-centric” perspective, behavioral tests tend to employ stimuli that share important features even when the exact methodology, intended purposes, or interpretations differ. For example, tests in which a dog is released to roam free in an empty testing area have recently been used to measure traits including activity level [e.g., (38)], independence [e.g., (39)], arousal and anxiety [e.g., (40)], or exploration tendency [e.g., (41)]. Irrespective of the intention of the testers, the experience of an empty room is likely to be similar for the participating dogs. For efficiency in discussion, it is practical to consider these tests together, but also to acknowledge the numerous possible contributors to behavior that might be reflected in a single test. As such, a “dog-centric” perspective may help us to put aside the subjective interpretations of behaviors, and instead consider the measures themselves. By identifying the methodological similarities and categorizing behavioral tests based on stimulus attributes, we offer an approach to consolidate the research across such a disparate field.

The current review sought to provide a practical starting point for researchers and practitioners seeking to select or design canine behavioral tests. To do so, we investigated the behavioral test stimuli used to measure canine psychological traits in the scientific literature. We aimed to create a framework that could parsimoniously describe the stimuli used in behavioral tests from a dog-centric perspective and, from this, review various methodological options and practical considerations for their application.

## 2 Methods

This review used a scoping review search method with a content analysis of the behavioral testing methods from the articles identified. We followed the Preferred Reporting Items for Systematic reviews and Meta-Analyses extension for Scoping Review (PRISMA-ScR) guidelines (42).

### 2.1 Eligibility criteria

We included peer-reviewed articles published in English that used behavioral testing to measure variation in psychological traits among dogs. Behavioral tests in this case included any standardized protocol in which a dog was presented with a stimulus and their responses recorded, and which did not require extensive specific training (> 1 week of training) for the dog to complete. Tests that analyzed the variation between individual dogs or groups of dogs were included and we excluded tests that analyzed at only a general species level (e.g., comparing dogs and wolves, or determining if dogs possess a cognitive ability). Finally, only tests that intended to measure psychological traits were included. The inclusion and exclusion criteria are specified in Table 1. In cases where an article used both in-scope test(s) and out-of-scope test(s), the article was included but only the in-scope test(s) were analyzed.

**Table 1:** Inclusion and exclusion criteria used to select articles.

Criteria	Inclusion	Exclusion
Article type	Experimental study published in a peer-reviewed journal	Review article Not published in a peer-reviewed journal (e.g., book, conference proceeding, thesis) Retracted publication
Language	Article available in English	Article available exclusively in a language other than English
Population	Participants were domestic dogs (*Canis familiaris*)	Participants were exclusively a species other than domestic dogs (*Canis familiaris*)
Methods	Reports a standardized behavioral test (i.e., measures were taken from direct observation of dogs responding to stimuli at a specific time point(s) using a protocol that is consistent across dogs)	Outcomes are only responses to a questionnaire Measure is from general observation of behavior, not at a specific time point(s) Test methods are not described or referenced Test measures performance of behavior(s) that required extensive protracted training (> 1 week)
Analysis	Individual differences were measured (i.e., scores from individual dogs or groups of dogs are differentiated)	Species-wide abilities or trends measured (e.g., only differentiations between species are reported)
Purpose of test	To measure or infer consistent tendencies or psychological traits	To measure physical or physiological differences (e.g., visual or olfactory acuity) To measure transient psychological states (e.g., fear during a surgical procedure) To measure the outcome of a short-term intervention, including training, interaction, pharmacological, nutraceutical, or hormonal interventions To measure clinical dysfunction or impairment To measure food preference or palatability To measure interactions or relationships within specific relationships (e.g., attachment style)

### 2.2 Information sources and search strategy

Search terms were initially chosen based on the terms used in titles, keywords, and abstracts of an initial selection (*n* = 71) of articles in the research area that were known to the research team. Following this, synonyms, plurals, and alternate spellings were added. Preliminary searches were conducted to check if known articles were included. To capture articles with different purposes and to retrieve a comprehensive sample of publications, the search terms were broad. However, since some reports use behavioral tests as part of their methods but do not mention this explicitly in their title, abstract, or keywords, it is acknowledged that the list of retrieved articles is likely not exhaustive.

Five databases (SCOPUS, Web of Science, CAB abstracts, PsycINFO, and Medline) were searched from their inception dates. The searches were first conducted on the 6th of January 2023 and updated on the 18th of March 2024. The following terms were used to search the titles, abstracts, and keywords of peer-reviewed journal publications: (dog OR dogs OR puppy OR puppies) AND (test OR task OR assessment OR measure* OR score* OR procedure OR protocol) AND (cognit* OR behavio* OR personality OR temperament OR character OR “problem solving” OR “problem-solving” OR fearfulness) AND (trait* OR characteristic OR factor OR ability OR differences OR suited OR suitability OR selection OR problems OR stability). Irrelevant subject areas were excluded from the search results (e.g., engineering, physics and astronomy, dentistry, economics).

Citations from the database searches were imported into the Covidence Systematic Review Software (43). Duplicates were automatically and manually removed. According to the exclusion criteria listed above, titles and abstracts were screened initially for relevance, followed by full-text screening of the remaining articles. The screening process was conducted by one reviewer (AM). A second reviewer was not deemed necessary to achieve our objective of a comprehensive, but not exhaustive, selection of publications, since high sensitivity and specificity of included articles were not necessary for the aims of this scoping review.

### 2.3 Data charting

All data were charted in a spreadsheet created for this purpose, which is supplied in the Supplementary materials. The data were extracted by manually reading each original article and recording the relevant features. The Supplementary materials present the list of categories and definitions for each feature.

For each article in the spreadsheet, the citation, author names, and year of publication were charted. We then categorized the general purpose of the behavioral test(s) in the article (e.g., research, shelter assessment, working dog assessment), extracted the terms used by the authors to describe the measured outcomes—referred to here as “trait descriptors”—and categorized these as relating primarily to the domains of behavior, cognition, or both.

Following this, methodological information was recorded, including the total number of participants, their age groups, and sources of studied dogs (e.g., companion, shelter), as well as the types of measures used (e.g., behavioral coding, hormonal assays). For each article, we counted the number of unique tests used (i.e., excluding repeated tests from the count). Articles using more than one test were considered to have used a test “battery” and, if applicable or available, the name or a brief description of the battery was recorded so that it could be referenced and compared across different articles.

We then recorded the names (or if not available, brief descriptions) of each behavioral test in a given article and whether each test was reported as having been adapted or replicated from another article in the dataset or was described in the first instance in this dataset. We charted the methodological detail for each test by extracting the relevant segments of the reporting article’s methods section. In cases where a test battery was replicated in its entirety and referenced as such, the relevant data were duplicated from the data charted for the original, referenced article.

Finally, we charted whether or not reliability (test–retest, intra-rater, or inter-rater) and validity (construct or criterion) metrics were reported in the article. This was recorded simply as presence or absence.

To report the number and type of trait descriptors used, we made a list of all the terms authors used to describe the outcomes measured by the test(s) that had been recorded in the spreadsheet, then counted the number of times that each trait descriptor (e.g., sociability, frustration) appeared. We then condensed the list of trait descriptors by manually combining all terms with the same word-stem and meaning (e.g., aggressiveness, aggression, and aggressivity). To avoid subjective interpretation, synonyms that did not share a word-stem were not combined (e.g., boldness was not combined with confidence) and specific terms were not combined with general terms (e.g., aggression towards dogs was not combined with aggression).

### 2.4 Content analysis

As a method of qualitative analysis, inductive content analysis can be used to understand and synthesize text-based data (44). This method involves reading the text, creating codes to describe each piece of its content, and then grouping these codes to synthesize the data into broad categories. The steps to conducting this analysis are (1) reading and familiarization with content, (2) first-round coding, (3) second-round coding, (4) redefining subcategories, and (5) synthesis and interpretation (44),

In the current study, the aim was to synthesize and present the patterns of canine behavioral testing methods in a framework that enabled us to discuss a large number of protocols. To do this, content analysis was used to investigate the test stimuli in each behavioral testing protocol. A stimulus was defined as any object or event used to elicit a behavioral response from a participating dog. We sought to describe and categorize the major stimulus or stimuli of each test procedure according to those which were most relevant to the responses measured in the test. Potential stimuli that were incidental, such as objects, sounds, or people that may have been in the area but were not the focus or intention of the test, were not coded.

We used a slightly modified process of content analysis appropriate for the breadth of methodological data that were analyzed. The first-and second-round coding processes were carried out with an initial subset of 20% of the included articles, which determined the first iteration of codes. Then, in the data charting spreadsheet, every article was coded by recording the presence or absence of each stimulus code. These data are supplied in the Supplementary materials. A test could be coded with more than one code in cases when more than one distinct stimulus was presented in a test simultaneously or successively. Throughout this process, the codes were redefined when appropriate. In this way, the codes were created iteratively and were developed and modified throughout the process until the test stimuli were considered to be adequately and parsimoniously described. Finally, the codes derived from this process were grouped into overarching categories for synthesis and discussion and each code was labelled and defined as a subcategory.

## 3 Results

**Figure 1:**
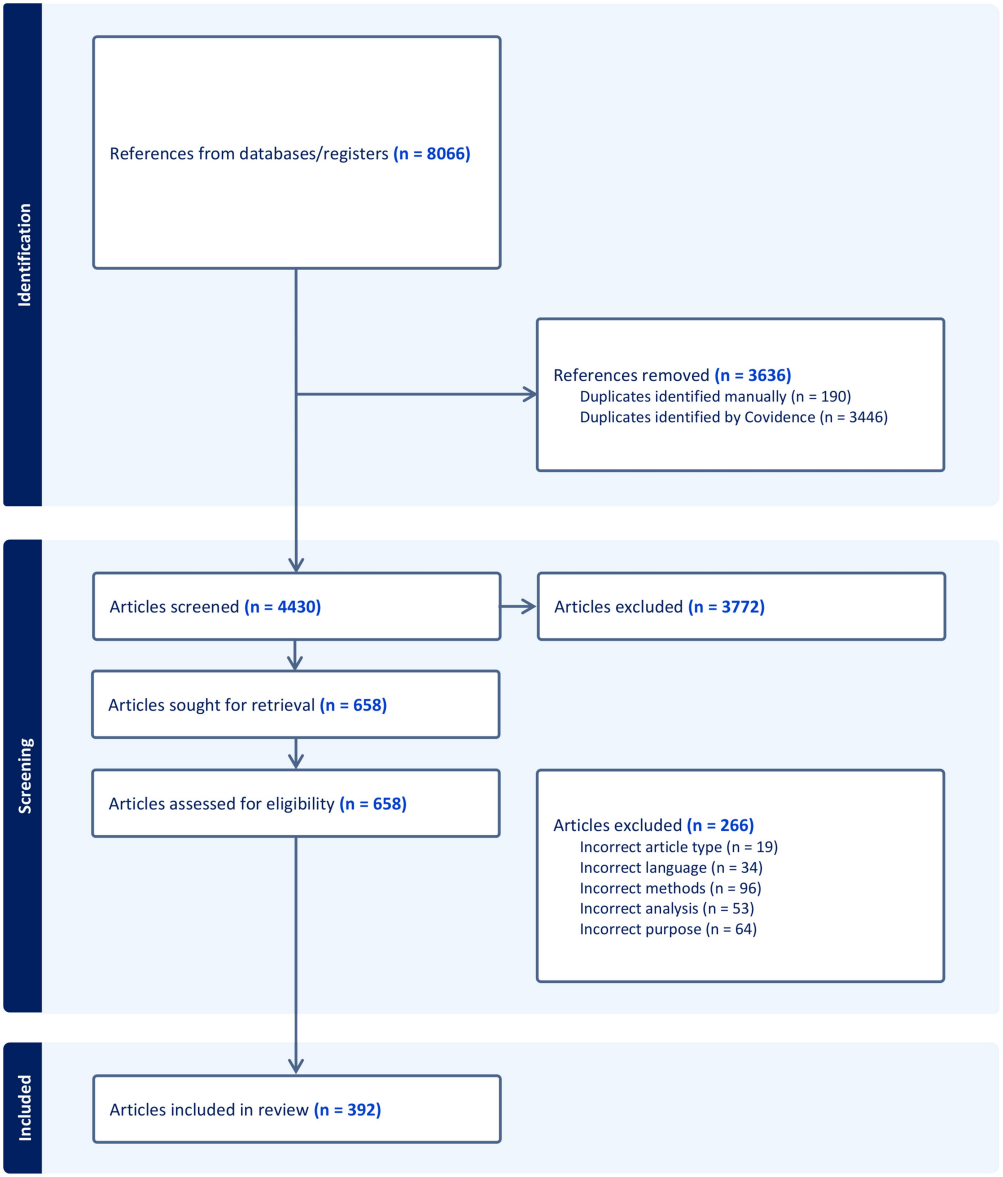
Preferred Reporting Items for Systematic Reviews and Meta-Analysis (PRISMA) flow chart for the present review.

Figure 1 shows the flowchart of the citation retrieval, screening, and inclusion process. From 4,430 unique citations, 392 were included in the review analyses. A complete list of the included articles is presented in the Supplementary materials. The articles were published between 1948 and 2024, with a majority (61.48%; 241/392) published in the most recent decade, from 2015 to 2024.

Most articles employed a battery of tests, with a mean of 6 unique tests per article (not including repeated tests) (SD = 6.02), ranging from 1 to 43. Overall, the reviewed articles included 2,362 behavioral tests in total. These included 326 that were adapted or modified and 982 that were replicated from other tests previously reported in the dataset. A further 1,054 tests were either described for the first time, replicated a test not previously appearing in the dataset or the scientific literature, or used a test without reference to its source in the text.

### 3.1 Characteristics of included articles

The complete data including all references and charted article characteristics are accessible in the Supplementary materials.

#### 3.1.1 Purpose of tests

Articles using behavioral tests for the primary purpose of research were most common in this dataset (68.62%; 269/392). These were articles that used behavioral tests with the primary aim of answering a research question to advance scientific understanding or to provide an instrument for research. The remaining articles reported tests used for purposes with specific applications separate from basic research. This included assessing behavior generally, often referred to as “temperament testing,” for companionship or breeding purposes (6.12%; 24/392). Another application was shelter testing (7.91%; 31/392), which sought to assess shelter dogs’ suitability for adoption as a companion, often with the aim of predicting potentially problematic behaviors post-adoption. Other articles assessed aggression specifically (3.83%; 15/392), for example, to determine if a companion dog is a risk to the community. Another important applied purpose was assessing dogs’ suitability to be used for various working roles, including assistance (5.87%; 23/392), military (2.81%; 11/392), detection (2.04%; 8/392), and other working roles (2.81%; 11/392).

In terms of the traits that were measured, 64.29% (269/392) of articles sought to measure traits related to behavior and 34.18% (154/392) of articles sought to measure traits relating to cognition, with an overlap of articles that measured traits related to both domains. The traits that the tests intended to measure were described with a wide range of terms, with 390 unique descriptors used among the reviewed articles. The most frequently used trait descriptors were: aggression (*n* = 58), fear (*n* = 48), sociability (*n* = 41), playfulness (*n* = 31), curiosity (*n* = 16), problem-solving (*n* = 15), fearlessness (*n* = 14), inhibitory control (*n* = 14), chase-proneness (*n* = 13), and anxiety (*n* = 11). Thirty-three articles did not label any traits and instead described the findings using direct behavioral descriptions (e.g., number of yawns, distance from stimulus) or a test performance score.

#### 3.1.2 Populations

The sample size used in each article ranged from 8 to 89,352 dogs (mean = 839.62, median = 70, SD = 5831.84). The distribution of sample size included outliers with very high sample sizes; the six highest sample sizes (>10,000 dogs) used data from the Swedish Dog Mentality Assessment project [described first in (15)] and the latest adaptation of this test (13).

Companion dogs (pet or family dogs) were used in 54.08% (233/392) of articles. Other populations, in order of most commonly to least commonly used, included candidate working dogs (dogs bred or selected to potentially perform a working role) (16.33%; 64/392), dogs in shelters (12.76%; 50/392), laboratory dogs (dogs owned by a research institution) (9.18%; 36/392), working dogs (dogs currently performing a working role) (9.18%; 36/392), kennel dogs (privately-owned dogs not kept for human companionship) (3.06%; 12/392), and free-ranging dogs (1.5%; 6/392).

Adult dogs (1 to 9 years old) were used in most articles (83.42%; 327/392) and, in many cases, juvenile (16 weeks to 12 months old) (25.26%; 99/392) and senior dogs (>9 years old) (29.08%; 114/392) were included in the same sample as adults. Puppies (<16 weeks old) were always differentiated as a separate sample from other age groups and generally administered different versions of behavioral tests, and were reported in 16.84% (66/392) of articles.

#### 3.1.3 Measures

We identified several different types of measures used to collect data from behavioral tests. Subjective rating, for which traits or behaviors were rated based on the rater’s interpretation of the behaviors (e.g., a rating of “fearfulness”) was used in 19.90% (78/392) of articles. Behavior rating, for which specific behaviors were defined and rated on a scale (e.g., a rating of jumping when greeting) was used in 32.14% (12/392) of articles. Behavior coding, for which specific behaviors were defined and coded based on presence/absence, duration, latency, or a similarly objective parameter, was most common, used in 64.03% (251/392) of articles. Hormonal concentrations were measured from biological samples (e.g., saliva, urine, blood) in 7.40% (9/392) of articles. Cardiac measures [e.g., heart rate, heart rate variability (HRV)] were used in 3.32% (13/392) of articles, accelerometer recordings of activity were reported in 0.77% (3/392) of articles, and other technologies (e.g., MRI imagining) were used in 0.51% (2/392) articles.

#### 3.1.4 Psychometric reporting

Psychometric indices relating to reliability and validity were reported in a minority of the articles. Indices of test reliability were reported in 39.54% (155/392) of articles, which included intra-rater reliability (2.30%; 9/392), inter-rater reliability (32.40%; 127/392), and test–retest reliability (10.46%; 41/392). Indices of test validity were reported in 32.91% (129/392) of articles. Construct validity was reported in 20.15% (79/392) of articles to indicate how well the test reflected a certain construct in comparison to other measures (e.g., a questionnaire measuring similar traits). Criterion validity was reported in 14.54% (57/392) of articles to describe how well the test measured or predicted an outcome (e.g., completion of training, success of adoption).

### 3.2 Content analysis of methods

The inductive content analysis process produced 29 codes to describe test stimuli; the codes assigned to each article are available in the Supplementary materials. The stimulus codes, hereafter referred to as subcategories, were grouped into three broad categories, labelled as human-oriented stimuli, environmental stimuli, and motivator-oriented stimuli.

#### 3.2.1 Human-oriented stimuli

This category describes test protocols that sought to elicit a response towards a human (Table 2; Supplementary Table S1). These test stimuli were the most commonly used in the current sample of the literature and have been included in test batteries for almost every applied purpose. This emphasizes the importance placed on dogs’ behavior towards humans specifically, which may help them to be safe and effective as companions and working dogs. Tests using human-oriented stimuli generally seek to measure traits described as sociability, aggression, fearfulness, playfulness, and obedience.

**Table 2:** Subcategories of human-oriented test stimuli and their descriptions.

Subcategory	Description
Indirect human encounter	One or more human(s) are in the area but are ignoring the participant dog and do not engage with them directly for the entire duration of the test.
Physical manipulation	A human physically manipulates the participant dog by grabbing, holding, and/or moving parts of the dog (i.e., more than merely patting or stroking.)
Obedience cues	A human provides verbal and/or non-verbal cues to perform a specific behavior, either after a brief training period (during the test) or with the assumption that the dog has learned that behavior previously. For example, “sit” or “come.”
Playful encounter	A human engages with the dog in a way that invites play, for example by playfully activating/engaging with an object.
Hostile human encounter	A human directly engages with the participant dog and/or handler in a way that is intentionally and explicitly aggressive, for example striking towards, or yelling at.
Human touching dog’s possession	The dog is given a possession, for example a food bowl or toy, and then a human reaches towards, touches, and/or moves the object. This may be done with an artificial hand (excluding playful encounter).
Human interaction	One or more human(s) interact directly with the dog in a manner not described in the previous categories (physical manipulation, obedience cues, a playful encounter, hostile encounter, or touching a possession). This is broad and includes a spectrum of behaviors from those that would be considered typical in companion dog-human interactions (e.g., approaching, petting, placing on leash) as well as unusual interactions (e.g., approaching and running away, wearing a sheet over body and banging broom on the ground).
Other human-oriented	A human-oriented stimulus in which there is no direct interaction and which does not constitute one of the other categories.

**Figure 2:**
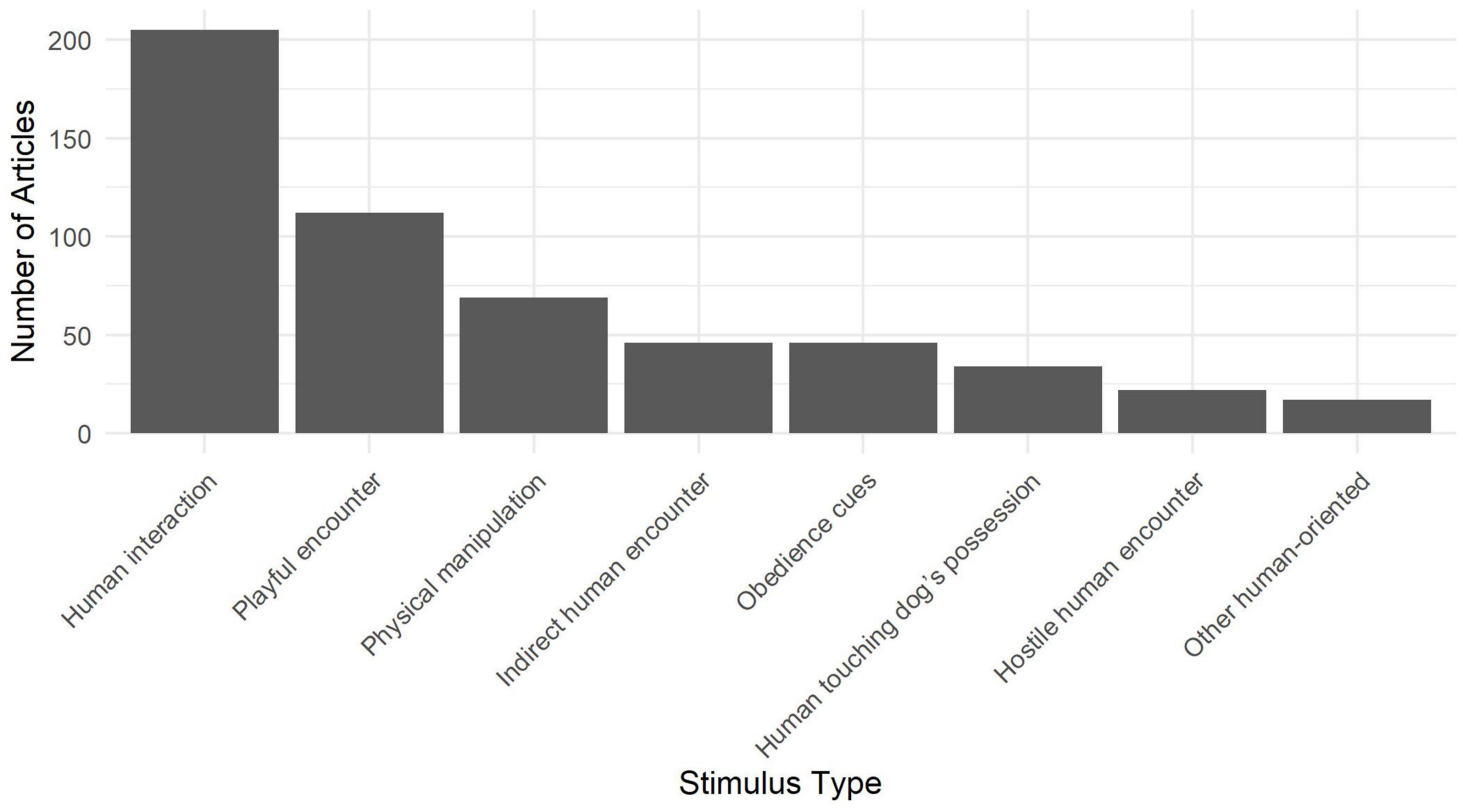
The number of articles that included one or more instances of each of the eight human-oriented stimulus subcategories.

While the contents of Table 2 are mostly self-explanatory, several points are of particular importance. First, by far the largest subcategory is Human Interaction (see Figure 2). This is a necessarily broad category to embrace the sheer scale of variation in tests involving human interactions. This category includes all protocols that involved a person interacting directly with a dog but that did not constitute the other, more specific, interaction categories (Physical Manipulation, Obedience Cues, Playful Encounter, or Hostile Encounter). These interactions include a spectrum of behavior from normal and sociable [e.g., calling the dog over and patting (45)], to the unusual and unexpected [e.g., wearing a hooded cape and approaching the dog while crouching and widening the cape (15)]. Different intensities of unusual behaviors may reveal different degrees of sociability, aggression, or fearfulness.

A second point of interest is that, in contrast to the stimuli that involve direct interactions, the subcategory Indirect Human Encounter refers to situations that allow testers to observe whether a dog chooses prosocial or antisocial behaviors while limiting the influence of the human leading the interaction. Additionally, Indirect Human Encounters often allow for the ability to manipulate the intensity of human behavior without it being directed towards or threatening the dog specifically [e.g., the “disgruntled stranger” test (46)]. This stimulus type was sometimes used to determine the safety of the dog with strangers [e.g., indicating if they will respond aggressively to people moving past (47)] but was more often used to indicate whether they seek out human contact [e.g., (23)], often as an initial step before beginning an interaction.

Third, Playful Encounters were used to reveal dogs’ motivation to play with humans and/or objects. This has been tested not only because it is a desirable trait for companion dogs (48), but also because it is critical for working dogs. In detection and military dogs (49, 50), for example, playfulness may be required to effectively train these dogs to carry out their roles.

Fourth, within each subcategory, the intensity of interactions and behaviors was often manipulated according to the purpose of testing. For example, if testers sought to determine if there was any potential for undesirable traits, such as aggression or fearfulness, human behavior that was particularly intense or challenging was often used to reveal this. In addition, in tests seeking to measure aggression, stimuli that could cause conflict, such as a Physical Manipulation or a Human Touching a Dog’s Possession, were used routinely [e.g., (51, 52)].

Studies have reported reasonable-to-excellent psychometric qualities for tests using human-oriented stimuli. Test batteries including human-oriented stimuli have found good test–retest reliability [e.g., (53, 54)]. Several studies have also established construct validity using owner reports of traits including aggression (55), fearfulness (56), sociability (57), activity-impulsivity (58), and other traits (13, 54). In addition, aggression tests have been able to differentiate dogs with a bite history from those without (59). In terms of criterion validity, evidence has been found for shelter tests using human stimuli to predict some behaviors of dogs following adoption, such as overall friendliness or fear (60), although they may not accurately predict undesirable behaviors, such as aggression (60–63). In addition, tests using human stimuli have been useful in predicting dogs’ success in assistance roles (64) and military roles (65).

#### 3.2.2 Environmental stimuli

The Environmental stimuli category includes stimuli that involve a setting, context, or the presentation of distinct objects or sensory stimuli (Table 3; Supplementary Table S2). These have been routinely used in test batteries for applied purposes, especially shelter tests, general temperament tests, and tests for working suitability, to observe whether dogs respond to the environment in ways that are adaptive and conducive to being successful in a companionship or working role. Tests using environmental stimuli tend to measure traits such as fearfulness, anxiety, boldness, reactivity, and activity level.

**Table 3:** Subcategories of environmental test stimuli and their descriptions.

Subcategory	Description
Unrestrained in empty area	The participant dog is let off leash with no human direction or interference in a testing area (not their usual home area), with no other purposeful test stimuli (note: If a human is present, they are not the focal stimulus and the dog had encountered them previously).
Unrestrained in area with stimulus options	The participant dog is let off leash with no human direction or interference in a testing area with multiple environmental stimuli.
Restrained in passive situation	The participant dog is restrained, such as with a leash or crate, in a testing area with no purposeful stimuli presentations.
Sudden visual stimulus	A visual stimulus is made to appear or change suddenly (i.e., quickly and unexpectedly).
Stationary object	A stationary object is presented.
Moving object	An object that moves, either on its own (e.g., robotic) or with human intervention (e.g., dragged on wheels), is presented. The object may become still after a period of moving.
Dog	One or more conspecific(s) is/are presented.
Animal	One or more non-human, interspecific animal(s) is/are presented.
Auditory stimulus	A loud or otherwise salient auditory stimulus is created in the vicinity of the participant dog.
Challenging surface or obstacle	An obstacle or surface (e.g., metal grate, stairs, wobbly surface) is presented.
Environmental walk	The participant dog is walked on leash by a person through a complex and naturalistic (i.e., non-controlled) environment.
Physical stimulus	A physical stimulus is applied to the participant dog.
Other environmental	An environmental stimulus which does not constitute one of the other categories.

**Figure 3:**
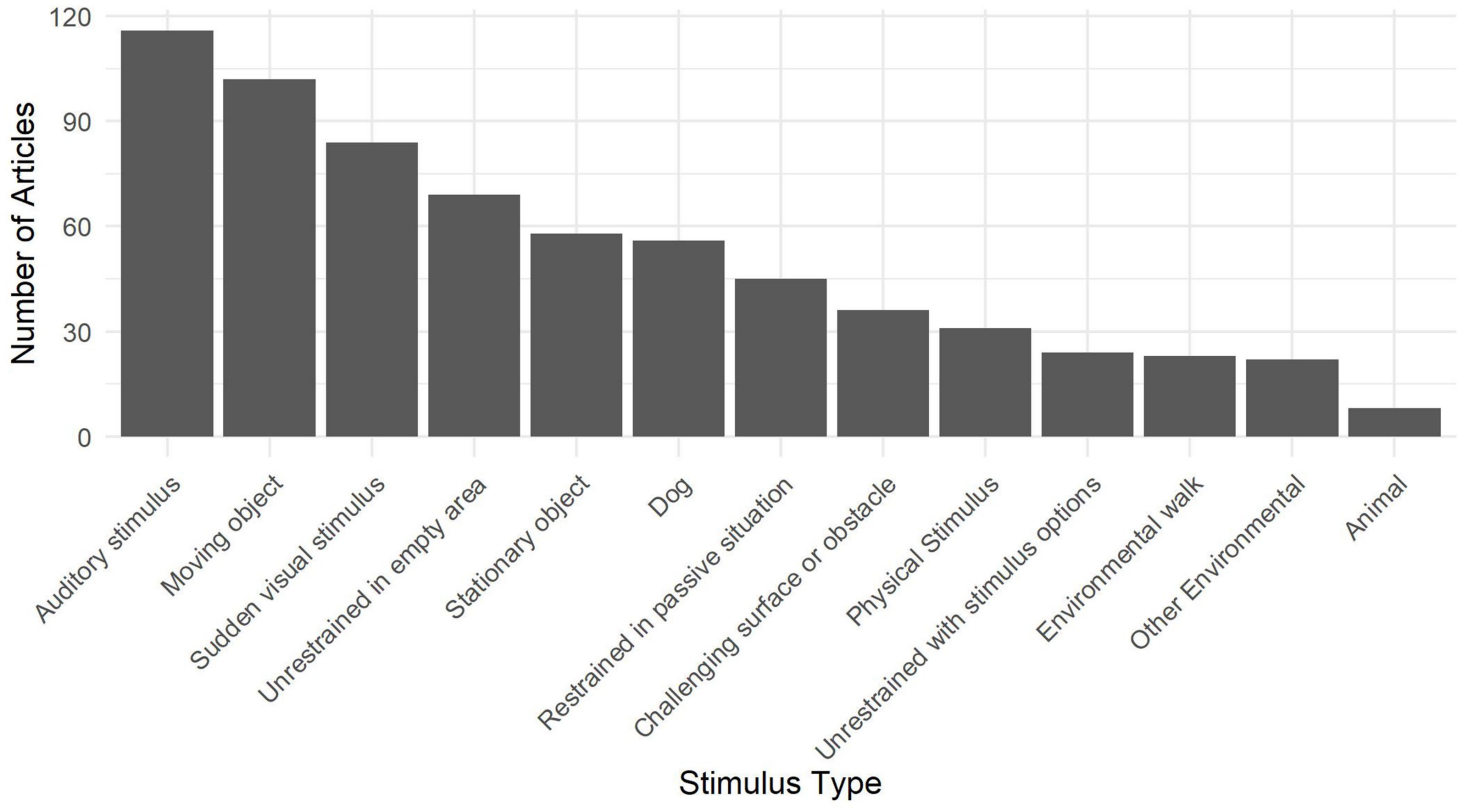
The number of articles that included one or more instances of each of the thirteen environmental stimulus subcategories.

The environmental stimulus subcategories are described in Table 3 and some additional points are of note. Firstly, the most commonly used stimuli in this category were Auditory Stimuli, Moving Objects, and Sudden Visual Stimuli (see Figure 3). These subcategories share a common theme of the stimulus presentations being particularly salient or startling. These challenging stimulus types were often used to potentially provoke behaviors that could reflect underlying problematic behaviors or undesirable traits, such as fearfulness and/or aggression (52, 66).

Secondly, the Stationary Object and Moving Object stimuli were often used with the intention of testing dogs’ response to novelty and, in particular, fear of novelty [e.g., (38, 67, 68)]. However, it was not reported how the novelty of these stimuli was ensured for most dogs. Nevertheless, many dogs predisposed to fear show fearful responses to objects that are familiar and so these may still be informative test stimuli even when novelty cannot be ensured.

In addition were objects that were used as proxies for humans, which are intended to gauge dogs’ potential aggressive responses to humans without risk of harm [e.g., (69)]. Although these tests are intended to measure a dog’s potential response to a human, rather than environmental stimuli, it is debatable whether dogs genuinely respond to these objects as humans. Nevertheless, their widespread use suggests that they have been useful to reveal extreme reactions and assess the risk of human contact.

Thirdly, contexts in which the dog’s free behavior could be observed, such as tests in which a dog is unrestrained in an area, with or without stimulus options, were often referred to in the animal behavior literature as open field tests [e.g., (6, 70, 71)] and arena tests [e.g., (24, 72, 73)]. These tests were also commonly predicated on the novelty of the area for the dog. In addition to observing a dog’s fear or boldness in a novel context, these tests were also used to observe a dog’s general activity and behavioral preferences, such as independently exploring the space, or being passive. However, a complicating factor in these tests was the presence or absence of a human; the presence of a human was often considered incidental in these tests, and sometimes not even reported. However, it has been demonstrated that the presence or absence of a human, especially a familiar guardian, has an impact on a dog’s exploratory or anxiety behavior (74). Conversely, in some cases, versions of these tests where no human was present were specifically referred to as “isolation” tests [e.g., (63, 75)].

Given the widespread importance of novelty for many of the environmental stimulus subcategories, determining test–retest reliability could be problematic in some cases. Some researchers have used different but comparable stimuli at each test time in an attempt to maintain novelty (49, 76). In cases when the time between testing is prolonged and there are not multiple instances of repeated testing, habituation to the stimulus is less likely and using the same stimuli may be appropriate [e.g., (54)].

Finally, studies using environmental stimuli have demonstrated construct validity by assessing concurrence between test behaviors and reports from owners and handlers, in particular for fearfulness (54, 56, 77). Others have found a link between test responses and physiological markers, such as salivary cortisol concentration (78, 79). Studies have also found criterion validity in predicting the likelihood of behavioral problems after adoption (80) or to become assistance dogs (81), detection dogs (82), military dogs (50), or guide dogs (83).

#### 3.2.3 Motivator-oriented stimuli

Motivator-oriented stimuli describe testing paradigms in which the participating dog is expected to respond to a test stimulus in an attempt to reach a resource (Table 4; Supplementary Table S3), which is typically defined as a physical target that the dog has a desire to attain. Food is considered an intrinsic motivator for all dogs and was therefore used as a motivator most commonly, while play objects were also used in some cases. In many protocols, dogs were tested initially to determine whether they were motivated to attain the target at the time of testing, for example by providing freely accessible food and observing whether the dog eats it. These tests tended to focus on traits such as motivation, persistence, problem-solving, and various other cognitive abilities or styles. Historically, motivator-oriented stimuli were used most often for basic research purposes, but there has been increasing use for applied purposes such as assessing working dog suitability (84).

**Table 4:** Subcategories of motivator-oriented test stimuli and their descriptions.

Subcategory	Description
Freely accessible food	Food is presented to the dog directly – it is freely accessible and they are able to consume it without interference or intentional distraction.
Navigate to reach motivator	A motivator is in the testing area and, in order to reach it, the participant dog needs to move towards it in a non-straightforward direction, for example around a barrier.
Manipulate object to reach motivator	A motivator is in the testing area and, in order to reach it, the participant dog needs to physically manipulate an object, for example by pushing open a container.
Inaccessible motivator	A motivator is presented but is physically inaccessible for the dog, for example within a container or out of reach.
Choice task	A motivator is placed in one of two or more discrete locations (e.g., buckets) and the dog may approach and check only one location. Information may be given about the location of the motivator, for example by a pointing gesture.
Reinforced behavior	A behavior is reinforced with a motivator so that the dog may learn to perform or inhibit a behavior.
Other motivator-oriented	A motivator-oriented stimulus which does not constitute one of the other categories.

**Figure 4:**
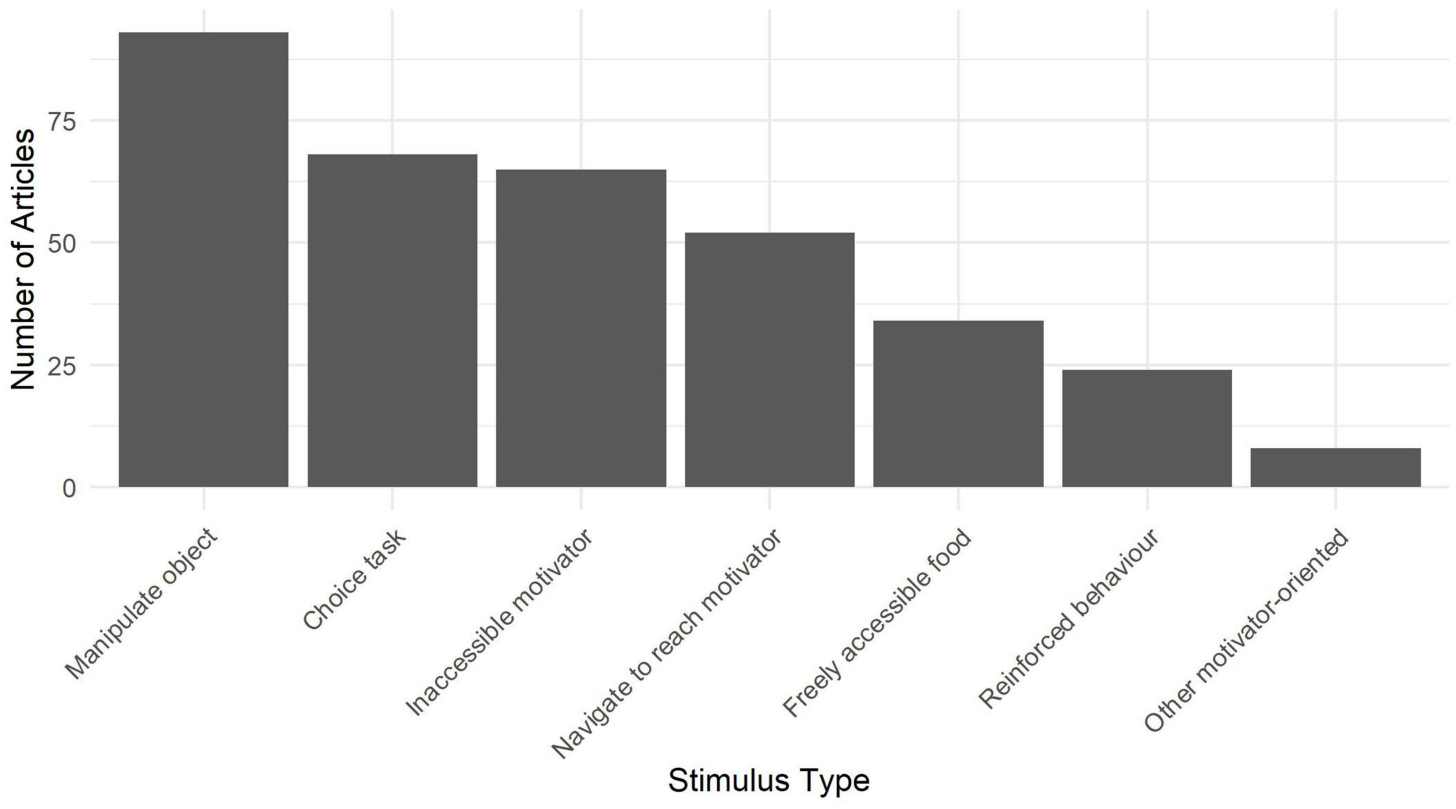
The number of articles that included one or more instances of each of the seven motivator-oriented stimulus subcategories.

Table 4 sets out the subcategories and descriptions of motivator-oriented stimuli and their use in testing. Notably, Manipulating an Object to Reach a Motivator was used most frequently in this category, followed by other problem-solving tasks including Choice Tasks and Navigating to Reach a Motivator (see Figure 4). In these tasks, the dog could reach the motivator if they performed the correct behavior(s), which was expected to reflect a particular ability. For example, tests that involved detouring around a barrier required the dog to inhibit the impulse of moving directly towards a motivator and instead to first move away from it to reach it, which may reflect their level of inhibitory control or impulsivity (7). In choice tasks, understanding information about where a motivator was hidden would allow them to reach it, for example by following a human’s pointing gesture, which may reflect their ability to understand human communicative cues (85). Also included were comparatively simple tests of manipulating an object to reach a motivator, such as extracting food from a tube or Kong™, to observe motor laterality or paw preference (86). Often, a test battery used many variations of the same paradigm, or stimulus type, to test different traits (87, 88).

In tests that used an inaccessible motivator, the dog was made aware that there was a resource out of reach, for example in a closed container. These were often referred to in the literature as an “unsolvable task” (89). This stimulus has been used most frequently to investigate interspecific social communication by observing whether dogs will make eye contact with a human when they are not able to reach the goal (90), as well as to reveal information about motivation, frustration, and persistence (91–93).

Tests involving reinforced behaviors trained the participant dog in a brief period to perform a behavior and then assessed traits related to learning, discrimination, and expectations. This was a diverse category that encompassed a variety of learned behaviors. An example of this stimulus type was “cognitive bias testing” that reinforced a dog for approaching an object when it was placed on one side of a testing set-up, but not when it was on the opposite side. They were then tested by placing the object in locations between these two sides, for which they had not yet learned the consequence. Latency to approach the object is taken as a proxy of optimism such that dogs approaching quickly are thought to be expecting reinforcement while those approaching slowly or not at all are not expecting reinforcement [e.g., (94)]. Similarly, in other reinforced behavior tests, the antecedents, behaviors, or consequences were manipulated to test various aspects of cognition.

In all subcategories, tests using motivator-oriented stimuli tended to facilitate the use of behavioral coding, which was advantageous for the intra-rater and inter-rater reliability of the measures. However, some tests may have issues with test–retest reliability due to the effect of learning. In cases in which learning is likely, researchers have reported high test–retest reliability for some tests (38) and low or mixed reliability for others (54, 95, 96). That said, overall, reporting of test–retest reliability was not common.

Since there are often no or very few established measures, such as questionnaires, for the cognitive traits that many motivator-oriented tests seek to measure, construct validity was often not established. However, some studies found good construct validity for traits with external measures, for example, impulsivity (95), and age-related cognitive decline (38, 97). In addition, criterion validity has been reported for motivator-oriented tests to identify suitable working dogs (84, 98–102).

#### 3.2.4 Test batteries

In the literature we collated, test batteries were used much more frequently than standalone tests. These allow for observation of an individual dog in a series of contexts, to determine consistent behavioral patterns, from which aggregating scores, such as average ratings or factor scores derived from a principal component analysis, could be used to estimate a trait. Furthermore, test batteries readily facilitate measuring more than one trait at a time, which is often desirable.

## 4 Discussion

This review systematically assessed 392 peer-reviewed articles, published from 1948 to 2024, that used behavioral testing with the aim of measuring individual differences in psychological traits in dogs. We sought to evaluate the extent of heterogeneity in methods and terminology in the field and then to find commonalities by categorizing and describing the stimuli used in testing protocols.

There has been a proliferation of canine behavioral testing literature since the last major review of the area (5), with over 60% of the articles included in the present review being published in the decade from 2015 to 2024. Many of the same issues, regarding heterogeneity in the literature, an overall lack of standardization in methods, reliability and validity reporting, and variable and often imprecise terminology, that were highlighted in previous reviews (4, 5, 29), persist. This may be unavoidable in such a broad research area, in which there has been considerable diversity in the purposes for employing tests, the sources and ages of participating dogs, and the measures used to collect data. These factors make it difficult, however, to compare and contrast tests such that they can be applied effectively and efficiently to address new research aims or applications.

Selecting the trait(s) to be measured is often the first consideration for researchers and practitioners when choosing appropriate behavioral tests. However, the terminology used to describe psychological and behavioral traits is inconsistent (4, 5). We found close to 400 terms that had been used to describe the traits or outcomes measured from behavioral testing. Often the same or similar testing protocols labelled the measured traits differently, sometimes due to researchers’ preferences and often due to the outcomes of factor analyses. Although efforts should continue to be made to clarify or standardize the terminology that is used to describe psychological traits, given the current state of the literature, it may be difficult to select appropriate behavioral tests based solely on a particular trait descriptor.

Another aspect that makes test selection difficult is the vast number of extant behavioral testing protocols; in this review, we found over 1,000 unique tests. To synthesize these findings, we compared the stimuli that were presented in testing protocols and found three major categories. First, tests with human-oriented stimuli presented dogs with humans either behaving neutrally or interacting with them at various levels of intensity (see Table 2). Second, tests with environmental stimuli presented non-human, sensory stimuli, including contexts, objects, odors, sounds, and physical sensations (see Table 3). Third, tests with motivator-oriented stimuli presented a tangible reward (food or play objects) to encourage the dog to engage in object-driven behavior (see Table 4). The subcategories within each of these three categories provide a reference for the types of canine behavioral tests reported and how they have been used. Knowledge of this structure may assist the selection, design, and use of behavioral tests in the future.

### 4.1 Practical considerations in testing

Our analysis of the literature, along with previous reviews of the field (4, 5, 29), highlights several important qualities that contribute to the practical use and accuracy of canine behavioral tests. We found that different categories of test stimuli had various advantages and disadvantages relating to these qualities.

#### 4.1.1 Standardization

Standardization of the testing protocol(s) within a study is important to ensure that variation measured among individual dogs cannot be attributed to variations in methods or stimuli. This is a particular risk with human-oriented stimuli, especially those involving direct interaction. Some of the potential factors that impact social interactions with dogs and are difficult to control include eye contact, voice tonality, physical movement (103), and odor, which are all likely to vary with changes in a person’s attributes, emotional state, or arousal level. Although some variation in human stimuli is unavoidable, this should be taken into account and particular care should be taken that the variation does not occur systematically according to the dog’s attributes. For example, people may behave more enthusiastically or affably with dogs they personally find endearing.

In addition, certain environmental stimuli risk excessive stimulus variation. In particular, Environmental Walks that expose dogs to non-controlled, naturalistic locations may feature considerable variation among the stimuli that the dogs encounter. In addition, when presenting other dogs or animals, care must be taken as far as possible to encourage the stimulus animal(s) to show standardized behavior. Studies that do not report their efforts at standardizing testing stimuli may be difficult to replicate and should attract caution when interpreting the results.

#### 4.1.2 Previous experience

In any behavioral test, it is relevant to consider the previous experiences that individual dogs may have had with a specific stimulus and how this might contribute to behavioral variation. For example, stationary and moving objects are often presented with the intention of testing dogs’ response to novelty [e.g., (38, 67, 68)], as novelty is a typical way for researchers to assess fearfulness in animals (72). However, this is not always straightforward in canine testing because, except for laboratory dogs who have often had controlled exposure to stimuli, it can be difficult to control whether a dog has had experience with the same or similar stimuli. For example, a flashing toy car (77) might be familiar to dogs in a family with children, but entirely novel for those from a household without children. As such, previous experience with a test stimulus may amplify or mask actual differences in psychological traits.

#### 4.1.3 Confounding effects

One of the drawbacks to behavioral testing is that it is susceptible to being influenced by state effects (transient variations in behavior) as well as other confounding factors, as opposed to purely measuring the trait or behavior of interest (5). One of the defining features of traits is that they are relatively stable over time, meaning that state and trait effects can be difficult to disentangle in data from a single snapshot in time. This can impact results for any test stimulus. For example, consider measuring the behavior of normally energetic and enthusiastic dogs on a day that they are unwell or fatigued. Repeated testing or a requirement to meet benchmark measures before proceeding with testing may ameliorate this issue, but it is difficult to avoid altogether in a behavioral testing context. Measuring and reporting test–retest reliability can indicate whether the test is sufficiently resilient against state effects, although this may be impossible for tests that require stimulus novelty.

Similarly, some of the variation in behavior observed in testing may be underpinned by characteristics other than what the test was intended to measure. For example, tests using motivator-oriented stimuli often seek to measure specific cognitive traits, but several other factors may affect dogs’ performance in these tests. Over the course of a test battery, dogs are likely to have different attention, motivation, and energy characteristics and therefore may show poorer performance in later tests. Similarly, although attempts are usually made to ensure that dogs are interested in the motivator, dogs will generally have different degrees of motivation for the reward and this can fluctuate based on their affective state and arousal (104). Where possible, it would be desirable to account for some of this variation, for example by benchmarking an individual dog’s test variables against their baseline, as well as designing protocols in a way that minimizes fatigue when resistance to fatigue is not a variable of interest for the test’s purpose.

#### 4.1.4 Multiple tests

As behavioral tests were most frequently administered in batteries of tests, it is necessary to consider individual tests in the context in which they were presented. The order in which behavioral tests are administered can impact dogs’ perception of and responses towards each stimulus due to experiences in the preceding tests. For example, the cumulative effects of multiple stressors in succession can elicit a stronger stress response, known as “trigger stacking” (105). This is particularly relevant for test batteries where the dog is presented with several challenging stimuli to assess traits such as aggression or fearfulness [e.g., (55)]. Similarly, as discussed previously, a series of motivator-oriented tests could diminish motivation over time. These carry-over effects can sometimes be useful and intentional, for example, eliciting a stronger response to a stressor to assess the potential for aggression (52). When compiling test batteries, one should consider possible carry-over effects from each test to the next and, in particular, how such effects may influence the validity of the data for the intended purpose.

Due to these carry-over effects, the reliability and validity statistics reported in studies with multiple tests are applicable primarily for the test battery, as a whole, and not necessarily the individual tests. Although these metrics provide an indication that a test is likely to be useful and accurate, it is possible that a test is valid in the context of a battery but elicits different results if administered in isolation. Therefore, accuracy should be assessed whenever a new test battery is used, even if individual tests are replicated or adapted from extant validated batteries. When appropriate, it is desirable to use an established test battery in its entirety to benefit from previous evidence of its validity.

#### 4.1.5 Welfare considerations

Welfare should be a priority in all human-dog interactions and the responsible use of behavioral testing should ideally improve welfare outcomes for dogs and people. Behavioral testing may improve canine welfare by informing behavioral and training interventions as well as the recruitment of dogs that are psychologically well-suited for their roles. Such interventions could minimize unnecessary stress throughout a dog’s lifetime.

Our review of the literature highlighted some important aspects relating to welfare in canine behavioral testing. Firstly, test stimuli that align with contemporary welfare standards should be selected by researchers and practitioners when designing and administering tests. Standards in welfare have changed over time and some of the test stimuli that were used in early canine behavioral tests [e.g., unpredictable electric shocks (12) and physically threatening or hitting dogs that have not been trained for protection (17, 106)] would be considered unethical today. Instead, behavioral testing should aim to be an enriching experience for participating dogs or, at a minimum, a neutral experience that does not cause any lasting psychological impact.

For some purposes, eliciting a degree of stress may be necessary to assess dogs’ behavioral responses to stress. For example, in tests of aggression or to determine suitability for high-arousal working roles. The invasiveness or challenge of a test should be balanced against the necessity or importance of the information that is collected, and the least invasive or stressful option should be chosen. It may be possible, for example, to use stimuli that are likely to elicit only short-term effects. It might also be possible to offer emotional comfort and relief following the presentation of a stressful test stimulus.

Improving the methodology of current canine behavioral tests may produce less invasive tests while still being informative. For example, instruments that facilitate the precise measurement of subtle responses, such as HRV [e.g., (107, 108)] or automated movement tracking [e.g., (109, 110)], may reveal variations that can predict responses to stress without directly eliciting a high-level stress response. Continuing to critically evaluate behavioral testing practices and improve testing methods aligns with the ongoing goal to improve welfare outcomes.

### 4.2 Limitations

There were limitations to the current review process. Since we reviewed only peer-reviewed articles and did not include grey literature or protocols from industry sources, the review has a bias towards behavioral tests used for research purposes specifically. Information about behavioral tests that are routinely administered in practical contexts may be underrepresented since they are only infrequently reported in the scientific literature. Although there were articles included in the dataset that reported tests created and used for applied contexts, most articles reported tests used for research purposes. Future research that focuses on behavioral assessments used commonly in industry applications would help to reconcile scientific and practical knowledge.

Qualitative content analysis, which was conducted in this study to categorize the test stimuli extracted from test protocols, is a subjective and interpretive process. The aim in this case was to broadly discuss and evaluate a large number of protocols. As such, the stimulus categories that were discussed could not reflect all of the nuance and variation among testing methods and, instead, reduced them to their central stimulus to be useful for discussion and general overview. For example, the broad subcategory of “human interaction” could be further dissected and explored with greater specificity to determine the subcategories of interaction types and their ability to elicit certain responses. When selecting a test protocol to replicate or adapt, it is necessary to consider the fine distinctions between protocols that may elicit behaviors or responses specific to that test. Additionally, there were some limitations in specificity for our categories, as many studies did not provide precise details of the testing protocol, meaning that some potentially important details were unknown. There also appeared to be some overlap between categories or differing potential perceptions of the most salient stimulus within a behavioral test.

Finally, the scope of this review was necessarily limited. We sought to discuss test stimuli and how they are used in canine behavioral testing, with the perspective that this is a critical aspect of test selection and design. Beyond the use of test stimuli, there are many other important factors to consider for behavioral testing that will impact the quality of emergent data, such as the selection of the participant population, the measures that generate data, and the interpretation and analysis of results. Several reviews discuss these other aspects of canine behavioral testing (4, 5, 27, 29).

## 5 Conclusion

The body of scientific literature that uses canine behavioral testing is immense and increasing rapidly. Many researchers and practitioners rely upon behavioral testing as a tool to investigate research questions and make practical decisions around assessing dogs’ suitability for roles. However, the field is vulnerable to issues relating to the standardization of methodology, terminology, quality reporting, and interpretation. This makes it difficult to critically evaluate, select, or design behavioral tests that are appropriate for an intended purpose. These difficulties could hamper the continued improvement of the methods used to assess canine behavior and cognition. The current review provides a comprehensive overview and practical reference of the methods and uses of published canine behavioral tests and offers a novel perspective by focusing on test stimulus categories. It is anticipated that this may help researchers and practitioners make informed decisions when choosing test protocols and interpreting responses from dogs.
