# Supplementary material for: Methods of behavioral testing in dogs: a scoping review and analysis of test stimuli
Source: Front Vet Sci. 2024 Oct 16;11:1455574. doi: 10.3389/fvets.2024.1455574 (PMC11521878; doi:10.3389/fvets.2024.1455574)
Supplement: Supplementary file 1 [file Table_1.docx]

**Supplementary Table 1. Subcategories of human-oriented test stimuli and their descriptions. Examples of brief test protocols and trait labels from articles using each stimulus type are included.**

| **Subcategory** | **Description** | **Brief protocol examples** | **Trait label examples** |
| --- | --- | --- | --- |
| Indirect Human Encounter | One or more human(s) are in the area but are ignoring the participant dog and do not engage with them directly for the entire duration of the test. | - Person ignores dog, remains seated  - Person walks around area ignoring dog  - Person stumbles near dog  - Person jogs in view of dog  - Person cycles in view of dog | Sociability  Aggression  Anxiety  Fear  Timidity  Dominance  Human aversion |
| Physical Manipulation | A human physically manipulates the participant dog by grabbing, holding, and/or moving parts of the dog (i.e., more than merely patting or stroking.) | - Person brushes dog  - Person touches feet, ears  - Person lifts up dog  - Person holds muzzle  - Person squeezes skin  - Person rolls dog onto side and holds | Aggression  Dominance  Submissiveness  Body sensitivity  Reactivity  Stress  Activity  Resistance  Sociability |
| Obedience Cues | A human provides verbal and/or non-verbal cues to perform a specific behavior, either after a brief training period (during the test) or with the assumption that the dog has learned that behavior previously. For example, “sit” or “come”. | - Sit, down, stand on cue  - Stay in position on cue  - Recall on cue  - Leave food on cue  - Lift paw on cue | Obedience  Trainability  Distractibility  Inhibitory control  Self-control  Laterality  Sociability  Inequity aversion |
| Playful Encounter | A human engages with the dog in a way that invites play, for example by playfully activating/engaging with an object. | - Person plays tug of war  - Person throws toy  - Person swings around toy  - Person starts playing tug and abruptly stops | Playfulness  Boldness  Temperament  Responsiveness  Arousal  Competitiveness  Object focus  Social referencing  Novelty seeking  Sociability-obedience |
| Hostile Human Encounter | A human directly engages with the participant dog and/or handler in a way that is intentionally and explicitly aggressive, for example striking towards, or yelling at. | - Person grabs dog’s face  - Person strikes at dog  - Person pretends to attack handler and dog  - Person threatens dog with stick  - Person hits dog with newspaper | Defense drive  Sharpness  Fearfulness  Aggression  Reactivity |
| Human Touching Dog’s Possession | The dog is given a possession, for example a food bowl or toy, and then a human reaches towards, touches, and/or moves the object. This may be done with an artificial hand. (Excluding playful encounter). | - Person takes food away while dog is eating  - Person takes bone away from dog  - Person takes toy away from dog | Resource guarding  Aggression  Sharpness  Possessiveness  Compliance  Friendliness  Disobedience  Dominance |
| Human Interaction | One or more human(s) interacts directly with the dog in a manner not described in the previous categories (physical manipulation, obedience cues, a playful encounter, hostile encounter, or touching a possession). This is broad and includes a spectrum of behaviors from those that would be considered typical in companion dog-human interactions (e.g., approaching, petting, placing on leash) as well as unusual interactions (e.g., approaching and running away, wearing a sheet over body and banging broom on the ground). | - Person approaches dog and looks at it  - Person enters enclosure  - Person speaks to dog  - Person squats and calls to dog  - Person claps towards dog  - Person pats dog  - Person clips leash to collar  - Person wearing sheet and gloves runs at dog  - Person wearing mask approaches dog  - Person wearing cape approaches  - Three people approach and surround dog  - Several people walk towards dog  - Person jumps towards dog | Sociability  Shyness  Boldness  Self-confidence  Social cognition  Fearfulness  Reactivity  Responsiveness  Affability  Friendliness  Aggression  Greeting behavior  Human focus  Social engagement  Approach behavior  Wariness  Timidity  Stress  Anxiety  Social motivation  Confident flexibility  Distraction |
| Other Human-Oriented | A human-oriented stimulus in which there is no direct interaction and does not constitute one of the other categories. | - Person knocking on and rattling car window  - Person wearing bite sleeve encourages dog to bite  - Owner hides and calls out to dog  - People act out social encounter, one is helpful to owner and one is unhelpful  - Training for leash walk (no extrinsic motivator) | Aggression  Attention  Sharpness  Activity  Social engagement  Friendliness  Showing behavior  Social evaluation |

**Supplementary Table 2. Subcategories of environmental test stimuli and their descriptions. Examples of brief test protocols and trait labels from articles using each stimulus type are included.**

| **Subcategory** | **Description** | **Brief protocol examples** | **Trait label examples** |
| --- | --- | --- | --- |
| Unrestrained in Empty Area | The participant dog is let off leash with no human direction or interference in a testing area (not their usual home area), with no other purposeful test stimuli.  (Note: If a human is present, they are not the focal stimulus and the dog had encountered them previously). | - Loose in unfamiliar room  - Loose in empty arena  - Loose in area and left alone | Independence  Activity  Excitability  Exploration  Active movement  Response to separation  Quiet investigation  Fearfulness  Anxiety  Arousal |
| Unrestrained in Area with Stimulus Options | The participant dog is let off leash with no human direction or interference in a testing area with multiple environmental stimuli. | - Arena with mirror, child’s toy, dam’s bedding  - Arena with empty side, partition, and side with balloon on string, cage with rat litter, clock, mirror  - Novel area with neutral human, ball, Kong™, rope toy, plastic disc  - Room with rag doll, ice cream container, rubber ball | Exploration  Activity  Fear  Anxiety  Reactivity  Emotional stability  Shyness  Boldness  Preference |
| Restrained in Passive Situation | The participant dog is restrained, such as with a leash or crate, in a testing area with no purposeful stimuli presentations. | - On leash in dark room  - Confined in crate  - Held on leash next to handler and a passive person  - Confined in car | Activity level  Excitability  Frustration  Reactivity  Separation anxiety  Dominance |
| Sudden Visual Stimulus | A visual stimulus is made to appear or change suddenly (i.e., quickly and unexpectedly). | - Umbrella opening and closing  - Slinky toy drops from ceiling  - Inflating balloon  - Mannequin suddenly pulled up to standing  - Mask on timber base suddenly raised up | Curiosity  Fearfulness  Shyness  Boldness  Nerve stability  Courage  Mental stability  Aggression  Reactivity  Recovery  Temperament  Timidity  Responsiveness  Confident flexibility |
| Stationary Object | A stationary object is presented. | - Rubber snake  - Crumpled newspaper  - Mirror  - Clown head  - Toy horse  - Dog statue  - Rocking horse  - Ball | Fearfulness  Reactivity  Confidence  Timidity  Object focus  Boldness  Nerve stability  Anxiety  Aggression  Distraction  Exploration |
| Moving Object | An object that moves, either on its own (e.g., robotic) or with human intervention (e.g., dragged on wheels), is presented. The object may become still after a period of moving. | - Mechanical snake slithering  - Flashing, moving toy car  - Ball rolled towards dog  - Small prey-like object dragged by cord in zigzag pattern  - Rolling ferret ball  - Flapping blanket  - Mechanical stuffed cat  - Child-sized doll on trolley wheeled toward dog | Chase-proneness  Boldness  Confidence  Object focus  Novelty seeking  Attentiveness  Temperament  Reactivity  Aggression  Fearfulness  Quiet investigation |
| Dog | One or more conspecific(s) is/are presented. | - Unfamiliar adult dogs in separate pens  - Unfamiliar dog walks into room  - Barking female dogs behind a fence  - Intact male dog behind a fence  - Dog given food next to participant dog  - Puppy in a wire cage  - Introduced to other dogs  - Cued to lift paw alongside another dog | Aggression  Fearfulness  Reactivity  Sociability  Resource guarding  Timidity  Confidence  Inequity aversion  Dominance |
| Animal | One or more non-human, interspecific animal(s) is/are presented. | - Cat in a cage  - In pen with chicken and goats  - Live bird released into area | Aggression  Response to novelty  Fearfulness  Reactivity  Retrieving ability |
| Auditory Stimulus | A loud or otherwise salient auditory stimulus is created in the vicinity of the participant dog. | - Doorbell sound  - Heavy object dragged  - Loud horn  - Shaking rattle  - Thunderstorm sounds  - Starting pistol  - Air horn  - Rattling metal cans  - Metal plate dropped onto concrete while dog is playing  - Gate slammed while dog eating food | Fearfulness  Shyness  Boldness  Gun shyness  Reactivity  Confidence  Mental stability  Aggression  Temperament  Reactivity  Timidity  Courage  Response to novelty  Neuroticism  Sound aversion |
| Challenging Surface or Obstacle | An obstacle or surface (e.g., metal grate, stairs, wobbly surface) is presented. | - Walked up flight of open steps  - Handler calls puppy over tunnel, stairway, A-frame  - Human walks upstairs, dog is loose  - Put on raised platform  - Bar that can only be jumped over | Fearfulness  Exploration  Environmental sureness  Motor laterality  Body sensitivity  Confidence |
| Environmental Walk | The participant dog is walked on leash by a person through a complex and naturalistic (i.e., non-controlled) environment. | - Walked through building  - Walked up and down a street  - Walked by highway with traffic  - Walked through busy shopping center | Fearfulness  Reactivity  Human focus  Docility  Environmental stability  Obedience  Aggression |
| Physical Stimulus | A physical stimulus is applied to the participant dog. | - T-shirt put on dog  - Boots put on dog  - Dog placed upside down in sling  - Adhesive tape on nasal bridge  - Blanket over head of dog  - Tea towel on dog’s back  - Electric shock | Reactivity  Aggression  Motor inhibition  Laterality  Fearfulness  Body sensitivity  Activity  Independence |
| Other Environmental | An environmental stimulus which does not constitute one of the other categories. | - Car journey  - Dog is put into box with light area and dark area  - Ball disappears behind screen and reappears as different size  - T-Maze (no motivator)  - Leash wraps around obstacle and wait for dog to untangle  - Video of dogs projected  - Dog investigates series of objects then novel object added  - Light turned off in room | Reactivity  Timidity  Confidence  Object permanence  Spatial abilities  Physical cognition  Novel object recognition |

**Supplementary Table 3. Subcategories of motivator-oriented test stimuli and their descriptions. Examples of brief test protocols and trait labels from articles using each stimulus type are included.**

| **Subcategory** | **Description** | **Brief protocol examples** | **Trait label examples** |
| --- | --- | --- | --- |
| Freely accessible food | Food is presented to the dog directly – it is freely accessible and they are able to consume it without interference or intentional distraction. | - Treat on ground  - Bowl of food  - Food reward on plastic plate at far end of pen  - Person offers food from hand  - Given rawhide chew | Food interest  Food motivation  Stress  Laterality  Coping |
| Navigate to reach motivator | A motivator is in the testing area and, in order to reach it, the participant dog needs to move towards it in a non-straightforward direction, for example around a barrier. | - Barrier directly in front of food, requiring detour to reach  - Fence between food and dog, person demonstrates walking around fence  - Retrieve food from one location several times then start location is changed  - Configurations of barriers in a maze  - Food is in cylinder that can only be accessed from the side | Food interest  Spatial problem solving  Spatial memory  Inhibitory control  Executive function  Physical cognition  Problem solving  Ability to learn from human demonstrator  Attention  Spatial cognition  Intelligence  Boldness  Learning  Memory |
| Manipulate object to reach motivator | A motivator is in the testing area and, in order to reach it, the participant dog needs to physically manipulate an object, for example by pushing open a container. | - Food in plastic cage that must be rolled over  - Food in puzzle toy  - Food can be reached by pulling rope under fence  - Food in plastic bin and person demonstrates how to open  - Food in metal can with small opening  - Retrieve food from under cup  - Kong™ toy with frozen food | Food motivation  Problem solving  Problem orientation  Social cognition  Physical cognition  Persistence  Human dependence  Response to novelty  Laterality  Impulsivity  Inattention |
| Inaccessible motivator | A motivator is presented but is physically inaccessible for the dog, for example within a container or out of reach. | - Food in wire mesh cage  - Food in plastic container attached to board  - Food in well in board with plexiglass cover  - Dog on lead and food thrown out of reach  - Toy held out of reach of dog while on lead | Problem solving strategy  Persistence  Social cognition  Social behavior  Communication  Frustration  Emotional arousal  Distraction  Food motivation |
| Choice task | A motivator is placed in one of two or more discrete locations (e.g., buckets) and the dog may approach and check only one location. Information may be given about the location of the motivator, for example by a pointing gesture. | - Increasing delays before dog is released to retrieve food from one of two boxes  - Toy visibly placed behind screen in one of three locations  - Food in two locations, person shows preference to one location  - Food hidden in one of two cups and person points to correct cup  - Food hidden repeatedly in one of two locations, then position swapped  - Food hidden in one of two containers and small blue block placed on correct one | Memory  Logical reasoning  Social cognition  Ability to follow pointing gesture  Attention  Inhibitory control  Intelligence  Decision speed  Impulsivity  Persistence  Object permanence |
| Reinforced behavior | A behavior is reinforced with a motivator so that the dog may learn to perform or inhibit a behavior. | - Dog is given food for making eye contact (gazing), then reinforcement ceases  - Dog receives food for reaching object in one location but not another location, then the object is moved to a central location between either side  - Dog is reinforced for pressing a buzzer to open a box and the buzzer is moved away from the box  - Dog is reinforced for waiting while low value reward is presented prior to high value reward is presented  - Dog given food for pressing lever then reinforcement ceases | Communicative learning  Tendency to gaze  Cognitive bias  Judgement bias  Optimism  Inhibitory control  Persistence  Trainability  Attentiveness  Discrimination learning  Executive function |
| Other motivator-oriented | A motivator-oriented stimulus which does not constitute one of the other categories. | - Dog is repeatedly called to step off stairs and approach experimenter and receives a food reward  - Person drops key to room with food in it, dog may help person retrieve key to access room | Laterality  Paw preference  Spontaneous cooperation  Attentiveness |
